# Cognitive Training Using a Novel Memory Game on an iPad in Patients with Amnestic Mild Cognitive Impairment (aMCI)

**DOI:** 10.1093/ijnp/pyx040

**Published:** 2017-07-02

**Authors:** George Savulich, Thomas Piercy, Chris Fox, John Suckling, James B Rowe, John T O’Brien, Barbara J Sahakian

**Affiliations:** 1 Department of Psychiatry (Dr Savulich, Mr Piercy, and Profs Suckling, O’Brien, and Sahakian), and MRC/Wellcome Trust Behavioural and Clinical Neuroscience Institute (Dr Savulich, Mr Piercy, and Profs Suckling, Rowe, and Sahakian) University of Cambridge, Cambridge, United Kingdom; Department of Psychological Sciences, University of East Anglia, Norwich, United Kingdom (Prof Fox); Department of Clinical Neurosciences and MRC Cognition and Brain Sciences Unit, University of Cambridge, Cambridge, United Kingdom (Prof Rowe).

**Keywords:** mild cognitive impairment, episodic memory, paired associates learning, cognitive training, motivation

## Abstract

**Background:**

Cognitive training is effective in patients with mild cognitive impairment but does not typically address the motivational deficits associated with older populations with memory difficulties.

**Methods:**

We conducted a randomized controlled trial of cognitive training using a novel memory game on an iPad in 42 patients with a diagnosis of amnestic mild cognitive impairment assigned to either the cognitive training (n=21; 8 hours of gameplay over 4 weeks) or control (n=21; clinic visits as usual) groups.

**Results:**

Significant time-by-pattern-by-group interactions were found for cognitive performance in terms of the number of errors made and trials needed on the Cambridge Neuropsychological Test Automated Battery Paired Associates Learning task (*P*=.044; *P*=.027). Significant time-by-group interactions were also found for the Cambridge Neuropsychological Test Automated Battery Paired Associates Learning first trial memory score (*P*=.002), Mini-Mental State Examination (*P*=.036), the Brief Visuospatial Memory Test (*P*=.032), and the Apathy Evaluation Scale (*P*=.026). Within-group comparisons revealed highly specific effects of cognitive training on episodic memory. The cognitive training group maintained high levels of enjoyment and motivation to continue after each hour of gameplay, with self-confidence and self-rated memory ability improving over time.

**Conclusions:**

Episodic memory robustly improved in the cognitive training group. “Gamified” cognitive training may also enhance visuospatial abilities in patients with amnestic mild cognitive impairment. Gamification maximizes engagement with cognitive training by increasing motivation and could complement pharmacological treatments for amnestic mild cognitive impairment and mild Alzheimer’s disease. Larger, more controlled trials are needed to replicate and extend these findings.

**Significance Statement**

Amnestic mild cognitive impairment (aMCI) has been described as the transitional stage between healthy ageing and dementia. aMCI is characterized by day-to-day memory difficulties and motivational problems. At present, there are no effective pharmacological treatments for the cognitive impairments of patients with aMCI. Cognitive training has shown benefits, but training packages are typically repetitive and boring. To maximize engagement with training, we developed a novel memory game in collaboration with patients with aMCI and tested its effects on cognition and motivation. We found that patients who played “Game Show” on an iPad for 8 hours over the course of 4 weeks showed improved episodic memory (memories of locations and events) compared with patients who attended clinic as usual. High levels of enjoyment and motivation were also maintained throughout all hours of gameplay. “Gamified” cognitive training can thus be used to enhance memory and motivation in patients with aMCI.

## Introduction

The number of people with dementia is 47.5 million worldwide, with 7.7 million new cases every year (World Health Organization, 2016). As innovations in the field of public health have lengthened the average human lifespan considerably, effective treatments for reducing cognitive decline in the aging population are needed. Cholinesterase inhibitors, including donepezil and rivastigmine, show modest time-limited benefits for individuals with Alzheimer’s disease (AD) but are more likely to be effective for the treatment of attentional dysfunction (Eagger et al., 1992; Sahakian et al., 1993). Since 2003, no additional treatments have been approved for AD, thus emphasizing the need for new interventions early in the course of the neurodegenerative process (Barnett et al., 2016). As such, alternative nonpharmacological strategies for memory restoration and enhancement are now being developed (Sahakian et al., 2015; Brühl and Sahakian, 2016; Savulich et al., 2017).

Individuals with amnestic mild cognitive impairment (aMCI), often the prodromal stage of AD, report mild short-term memory difficulties but preserved independence in activities of daily living (Albert et al., 2011). Individuals with multiple domain MCI can also show subtle decline across a range of other cognitive processes including language, attention, executive functions, problem solving, processing speed, and visuospatial abilities (Petersen, 2011). The prevalence of MCI is estimated to be around 3% to 20% of individuals above the age of 65, and at least 10% to 15% of these individuals progress to AD annually (DeCarli, 2003). A significant proportion of individuals with MCI also experience comorbid neuropsychiatric symptoms, such as apathy, anxiety, and depression, which negatively impact treatment entry and engagement and significantly predict conversion to AD (Monastero et al., 2009).

There are currently no approved drugs by the US Food and Drug Administration, European Medicines Agency, or Medicines and Healthcare products Regulatory Agency for the treatment of cognitive dysfunction, including memory impairment, in individuals with MCI. Previous clinical trials have failed to show any pharmacological intervention that slows progression of MCI to AD (e.g., Petersen et al., 2005; Feldman et al., 2007; Winblad et al., 2008). Cognitive remediation strategies, including cognitive training, are now used to enhance memory in neuropsychiatric disorders, with positive effects found in patients with schizophrenia (Wykes et al., 2011). Cognitive training is also effective in MCI (for reviews, see Belleville, 2008; Jean et al., 2010; Martin et al., 2011; Gates et al., 2011; Reijnders et al., 2013). Specifically, individuals with MCI receive mild-to-moderate benefits in episodic, semantic, and working memory, as well as in language, attention/processing speed, self-rated anxiety/depression, visuospatial abilities, functional abilities, activities of daily living, and quality of life (Li et al., 2011). Cognitive training of memory has also been shown to increase activation and connectivity in widespread frontal, temporal, and occipital regions of the brain (Sanz Simon et al., 2012; Strenziok et al., 2014), including the hippocampus (Rosen et al., 2011; Kirchoff et al., 2012), in healthy older adults and in patients with MCI.

Despite the advantages of cognitive training, one major challenge is overcoming the high incidence of dropout rates typical of patient groups. The cost and inconvenience of delivery (e.g., use of specialized equipment, participant travel), in addition to apathetic or depressive symptoms often characteristic of older populations with memory difficulties, leads to considerable dropout rates. Engagement with cognitive training programs may therefore require a more motivational approach by increasing task-related enjoyment.

Computer games are highly rewarding and can deliver targeted cognitive training programs that also have the potential to improve motivational deficits found in patient groups with severe neuropsychiatric symptoms (Savulich et al., 2017). Previously, video game training has shown to enhance sustained attention and working memory in older adults aged 60 to 85, with gains persisting up to 6 months and similar to those observed in untrained 20-year-olds (Anguera et al., 2013). We recently demonstrated that 8 hours of playing “Wizard” (www.peak.net/advanced-training), a memory game designed for patients with schizophrenia, improved episodic memory and functional outcome compared with a treatment-as-usual group (Sahakian et al., 2015). Importantly, those that played our game reported high levels of enjoyment and desire to continue after each hour of gameplay, which likely represents a motivational improvement over traditional (usually boring) cognitive training programs. Gamified cognitive training can simultaneously improve cognition and motivation, and thus comprises an appealing option for disorders in which pharmacological treatments have failed to treat cognitive dysfunction or do not address the associated motivational deficits.

The present study aimed to test the effects of “Game Show,” a novel learning and memory game, on cognition and motivation in patients with aMCI. We hypothesized that patients in the cognitive training group (8 hours of supervised gameplay on an iPad over the course of 4 weeks) would demonstrate improved episodic memory, our key trained domain, and visuospatial abilities, a secondary measure of near transfer, compared with patients in a control group (clinic visits as usual). We further hypothesized that the cognitive training group would maintain high levels of self-reported enjoyment and motivation throughout all hours of gameplay.

## Methods

### Participants

Forty-two patients were recruited from the Dementias and Neurodegeneration NIHR Clinical Research Network (Eastern DeNDRon) and memory clinics at the Cambridge and Peterborough NHS Foundation Trust, Cambridge University Hospitals NHS Foundation Trust, and Norfolk and Suffolk NHS Foundation Trust. All patients had MCI by NIA AA core clinical criteria for MCI of the AD type (Albert et al., 2011), as confirmed by their referring clinician prior to study enrollment. Exclusion criteria were a current or past neurological disorder or a current neuropsychiatric disorder affecting memory. Only adults aged 45 years and older with normal to corrected vision were invited to participate. All participants gave written informed consent.

### Neuropsychological Assessment

The National Adult Reading Test (Nelson, 1982) is a widely used estimate of premorbid intelligence (full-scale IQ range 69–131).

### Neuropsychiatric Symptoms

#### Geriatric Depression Scale (GDS Short Form)

The GDS (Sheikh and Yesavage, 1986) is a 15-item measure of depression in the elderly. Participants answer yes/no questions, with a score ≧5 suggestive of depression and a ≧10 almost always indicative of depression (range 0–15).

#### Hospital Anxiety and Depression Scale (HADS)

The HADS (Zigmond and Snaith, 1983) is a 14-item measure of anxiety and depression. Each item is scored from 0 to 3, with a higher total score indicating a higher level of anxiety and depression.

#### Apathy Evaluation Scale (AES)

The AES (Marin, 1996) is an 18-item measure of apathy. Participants are instructed to indicate how true each statement is of them in the past couple of weeks (from 0 = not at all to 3 = very). Higher scores indicate greater levels of apathy, with a score of 43 or greater usually indicating clinically significant apathy (range 0–54).

### Cognitive Measures

#### CANTAB Paired Associates Learning

The Cambridge Neuropsychological Test Automated Battery Paired Associates Learning (CANTAB PAL) assesses episodic memory and new learning and is a commonly used and highly sensitive test for MCI and early AD (Sahakian et al., 1988; Swainson et al., 2001; Blackwell et al., 2004; Barnett et al., 2016). Using a touch-sensitive computer screen, boxes are displayed and opened in a randomized order. One or more of them will contain a pattern. The patterns are then displayed in the middle of the screen, one at a time, and the participant must touch the box where they think the pattern was originally located. If the participant makes an error, then the patterns are re-presented to remind the participant of their locations. The level of difficulty increases throughout the task, with 1-, 2-, 3-, 6-, and 8-pattern stages. In line with previous studies (e.g., Sahakian et al., 1988, 2015; Kaser et al., 2016), outcome measures include total errors adjusted, total trials adjusted, and first trial memory score (i.e., the number of patterns correctly located after the first trial summed across the number of stages completed).

#### Mini-Mental State Examination (MMSE)

The MMSE (Folstein et al., 1975) is a 30-point measure of cognitive impairment that is commonly used to screen for dementia. Possible points for each category of assessment are as follows: orientation to time (5), orientation to place (5), registration (3), attention and calculation (5), recall (3), language (2), repetition (1), and complex commands (6). Scores indicate severe (<10), moderate (10–17), or mild (18–23) dementia.

#### Brief Visuospatial Memory Test-Revised

The Brief Visuospatial Memory Test-Revised (BVMT-R) (Benedict, 1997) is a measure of visuospatial memory. In the learning trial, participants view a stimulus page of 6 geometric figures for 10 seconds and are asked to draw as many of the figures as possible, without looking, in their correct location. Participants are given 3 opportunities to do this. A delayed recall trial is then administered after 25 minutes. Participants are then asked to identify in a forced choice recognition trial which of 12 figures were included among the original geometric figures (indicating yes/no responses). Finally, a copy trial is administered to screen for severe visuoconstructive deficits and to help score recall responses. Task versions were counterbalanced across testing sessions. A maximum of 12 points can be awarded for each trial based on the accuracy and location of the drawings. Measures of total recall (Trial 1 + Trial 2 + Trial 3 raw scores; range 0–36, higher scores indicating better visuospatial learning and memory), learning ([higher of trial 2 or trial 3] – trial 1 raw scores; range 0–12, higher scores reflecting more learning over successive presentation trials), delayed recall (range 0–12, higher scores indicating better recent, long-term visuospatial memory), and percentage retained ([delayed recall/(higher of trial 2 and 3)] x 100; range 0–100, higher scores indicating more information originally learned that was retained across the delay interval) are calculated.

#### CANTAB Choice Reaction Time (CRT)

The CANTAB CRT assesses the participant’s ability to react as quickly as possible to basic stimuli. It is used to assess motor speed and thus acts as a control measure of general alertness to help interpret other cognitive tasks. An arrow will appear on either the left or right side of a computer screen. After the arrow appears, the participant is instructed to press a corresponding left or right button, using a response box, as quickly as possible.

#### Game Show

Game Show was developed in collaboration between healthy older adults, patients with aMCI, a cognitive experimental psychologist, and a professional games developer. Our target cognitive process for improvement was episodic memory. This was decided from previous studies in our laboratory showing neuropsychological evidence of impaired memory performance (Swainson et al., 2001; Blackwell et al., 2004) and neuroimaging evidence of hippocampal dysfunction (de Rover et al., 2011) during the CANTAB PAL task in patients with MCI.

Game Show was designed using visually appealing displays and stimulating music. Pilot data was collected from 16 individuals (12 healthy elderly individuals and 5 patients with aMCI) to ensure that our game was fun, motivational, easy to understand, and in line with a concept suitable for an older adult population (i.e., daytime television game shows). Individuals played Game Show on an iPad for up to 1 hour and provided feedback on their experience, with suggestions for improvement. High levels of enjoyment and motivation were prioritized during development, as these properties were the key advantages of our game over other cognitive training programs. Pilot data from patients with aMCI indicated that the final version of our game was highly enjoyable (M = 9.32, SD = .66) and motivational (M = 9.40, SD = .58) (rated using 10-cm visual analogue scales, with higher scores indicated greater experience of enjoyment and motivation).

#### Procedure

**Figure 1.**
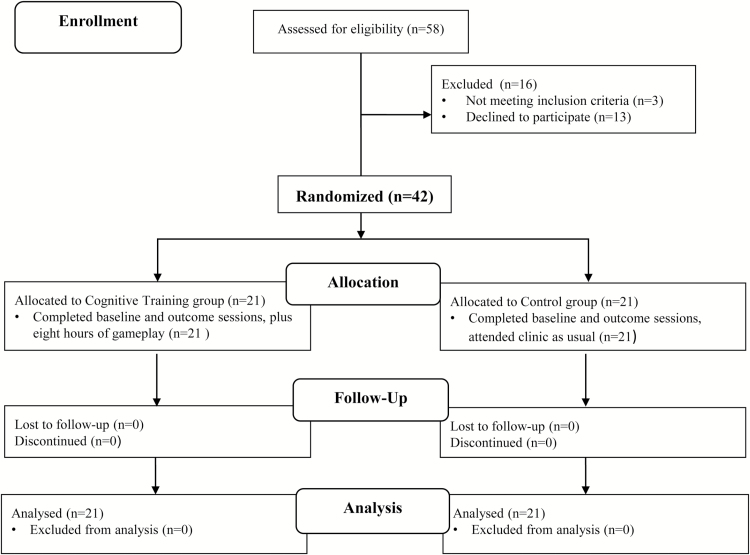
Consolidated Standards of Reporting Trials (CONSORT) flow diagram depicting passage of participants through the randomised controlled trial.

This study received full ethical approval from the National Research Committee East of England-Norfolk (13/EE/0290). The study comprised a 2-group, randomized controlled design (see Figure 1 for a Consolidated Standards of Reporting Trials flow diagram depicting the passage of patients through the randomized controlled trial; Moher et al., 2001). Invited patients were randomly assigned to either the cognitive training or control group.

Participants attended a baseline testing session in which they provided basic demographic information and were asked about their technology use, including the amount of time spent each week browsing the Internet and/or playing computer games and their confidence using new technological devices. Participants were also asked if they were currently playing any cognitive training games or applications. Participants were asked to complete computerized and pen-and-paper assessments of cognition and questionnaire assessments of neuropsychiatric symptoms.

Participants in the cognitive training group were invited to attend eight 1-hour sessions of supervised cognitive training (i.e., play Game Show on an iPad). During cognitive training, the player takes part in Game Show to win gold coins. In each round, the player is challenged to associate different geometric patterns with different spatial locations. Each correct answer allows the player to earn more coins. Rounds continue until completion or after 6 attempts are made. The number of geometric patterns presented is titrated depending on the player’s performance to keep users motivated and engaged. A “host” encourages the player to maintain and progress beyond their last played level. After each hour of gameplay, participants rate their experience in terms of enjoyment, desire to continue, level of self-confidence, and self-rated memory ability using 10-cm VAS. Participants in the control group were not given access to Game Show and attended clinic as usual.

At a maximum of 4 weeks after the baseline testing session, all participants completed an outcome testing session, which was identical to the baseline testing session in terms of outcome measures.

### Statistical Analyses

Demographic and baseline questionnaire measures were analyzed using independent samples *t* tests or Mann-Whitney U tests for continuous variables and chi-square for categorical variables. Cognitive and questionnaire measures (dependent variables combined) were first measured at baseline and outcome separately using multivariate ANOVA to reduce the probability of making a Type 1 error. Posthoc repeated-measures ANOVAs were then performed on each dependent variable, with time (baseline, outcome) as the within-subjects factor and group (cognitive training, control) as the between-subjects factor. Total PAL errors and trials were analyzed with an additional within-subjects factor of pattern (1-, 2-, 3-, 6-, and 8-patterns) to determine the effects of gameplay at different levels of task difficulty. Independent samples *t* tests were used to interpret significant interactions by determining differences between groups for each measure at baseline and outcome. Paired samples *t* tests were also used in each group separately to isolate the effects of cognitive training from baseline to outcome.

## Results

### Demographic Variables

The cognitive training (n=21) and control (n=21) groups did not differ in basic demographic variables including age, gender, premorbid intelligence, years in education, technology use (Internet and game playing), and confidence using new technological devices (all *P*>.42). The 2 groups also did not differ in baseline global cognition, depression, and apathy (all *P*>.31) and scored within the nondementing range of the MMSE (Table 1). No participants were currently playing any cognitive training games or applications.

**Table 1.** Groups Were Matched on Basic Demographic Variables, Baseline Technology Use, Global Cognition, and Neuropsychiatric Symptoms

	Cognitive Training Group (n=21)	Control Group (n=21)	Statistics
Age (y)	75.2 ±7.4	76.9 ±8.3	*t*(39.51)=-.67, *P*=.50
Intelligence (NART)	110.2 ±7.1	112.2 ±8.9	*t*(40)=-.81, *P*=.43
Gender (male, female)	11 M, 10 F	14 M, 7 F	*X* ^2^=.89, *P*=.53
Age left education	15.9 ±1.3	16.0 ±2.1	*U*=201.50, *P*=.62
Internet use (hours/week)	2.2 ±6.6	2.3 ±4.5	*U*=215.0, *P*=.87
Computer gameplay (hours/week)	.9 ±2.1	.7 ±1.9	*U*=201.0, *P*=.47
Confidence using new technology	11 Very confident 2 Confident 3 Apprehensive 4 Very apprehensive	13 Very confident 4 Confident 1 Apprehensive 3 Very apprehensive	*X* ^*2*^ *=* *2.33, P*=.*51*
Global cognition (MMSE)	26.6 ±2.9	26.8 ±2.2	*t*(40)=-.24, *P*=.81
Depression (GDS)	4.1 ±3.6	3.3 ±1.9	*t*(30.32)=.87, *P*=.39
Anxiety/Depression (HADS)	7.2 ±5.1	7.1 ±4.1	*t*(38.16)=.13, *P*=.90
Apathy (AES)	16.2 ±10.8	19.1 ±7.4	*t*(40)= -1.0, *P*=.32

Abbreviations: AES, Apathy Evaluation Scale; GDS, Geriatric Depression Scale; HADS, Hospital Anxiety and Depression Scale; MMSE, Mini-Mental State Examination; NART, National Adult Reading Test.

### Multivariate ANOVA

There was a significant difference in dependent variables (PAL total errors adjusted, PAL total trials adjusted, PAL first trial memory score, MMSE score, GDS score, AES score, BVMT-R total recall, BVMT-R learning, BVMT-R delayed recall, and BVMT-R percentage of information retained) at the group level at outcome [*F*(10,28)=3.78, *P*=.003, Wilk’s Λ=.43, η_p_^2^=.57], but there was no significant multivariate effect at the group level at baseline [*F*(10,29)=1.37, *P*=.24, Wilk’s Λ=.68, η_p_^2^=.32]. Furthermore, the time-by-group interaction was significant [*F*(9,29)=3.19, *P*=.008, η_p_^2^=.50].

### Repeated-Measures ANOVA

#### CANTAB PAL

##### Errors.

**Figure 2.**
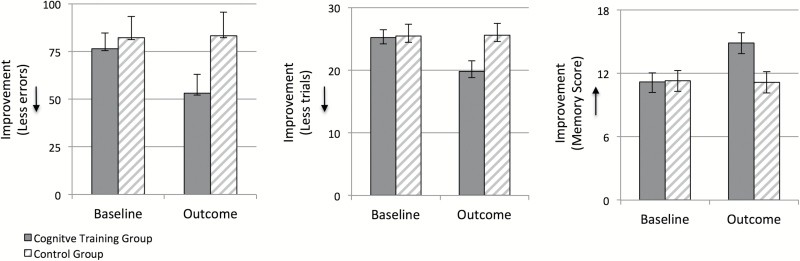
The cognitive training group made fewer errors, needed fewer trials, and had improved first trial memory score on the Cambridge Neuropsychological Test Automated Battery Paired Associates Learning (CANTAB PAL) task from baseline to outcome.

The time-by-pattern-by-group interaction for total errors adjusted was significant [*F*(4,37)=2.72, *P*=.044, η_p_^2^=.23] with main effects of time [*F*(1,40)=5.77, *P*=.021, η_p_^2^=.13] and pattern [*F*(4,37)=25.83, *P*<.001, η_p_^2^=.74] (Figure 2). Follow-up independent samples *t* tests showed that the cognitive training group made significantly fewer errors at the second- and third-pattern stages compared with the control group at outcome [*t*(21.68)=-2.40, *P*=.025, d=.13 and *t*(24.87)=-2.07, *P*=.049, d=.64, respectively] (Table 2). The number of errors made between groups at the 6-pattern stage at outcome did not reach significance [*t*(40)=-1.86, *P*=.071]. Paired samples *t* tests showed that the number of errors made from baseline to outcome was significantly reduced at the second [*t*(20)=3.13, *P*=.005] and third [*t*(20)=2.28, *P*=.034] pattern stages in the cognitive training group, but not the control group (*P* >.24). Total errors were also significantly reduced within the cognitive training group [*t*(20)=3.20, *P*=.005], but not the control group [*t*(20)=-.17, *P*=.86].

**Table 2.** The Number of Errors Made and Trials Needed by Group at Each Level of Pattern Difficulty on the CANTAB PAL Task at Baseline and Outcome

	Baseline	Statistics	Outcome	Statistics
Errors				
One-pattern	CT: .05 ±.22 CON: .19 ± 0.51	*t*(27.04)=-1.78,*p*=.25	CT: .00 (.000) CON .00 (.000)	–
**Two-pattern**	CT: 1.29 ± 1.45 CON: 1.81 ± 3.23	*t*(40)=-.68, *p*=.50	**CT: .33 ± .73** **CON: 2.24 ± 3.56**	***t*(21.68)=-2.40,*p*=.025**
**Three-pattern**	CT: 6.76 ± 5.50 CON: 8.19 ± 10.32	*t*(40)=-.56 *p*=.58	**CT: 3.24 ± 5.28** **CON: 10.43 ± 15.03**	***t*(24.87)=-2.07,*p*=.049**
Six-pattern	CT: 21.33 ± 15.68 CON: 27.62 ± 19.05	*t*(38.57)=-1.17,*p*=.25	CT: 17.38 ± 15.47 CON: 27.10 ± 18.34	*t*(40)=-1.86 *p*=.071
Eight-pattern	CT: 47.00 ± 24.33 CON: 44.38 ± 27.24	*t*(40)=.33 *p*=.74	CT: 32.19 ± 28.80 CON: 43.43 ± 27.67	*t*(40)=-1.29 *p*=.21
Trials				
**First trial memory score**	CT: 11.19 ± 3.9 CON: 11.29 ± 4.5	*t*(40)=.07 *p*=.94	**CT: 14.9 ± 4.5** **CON: 11.1 ± 4.7**	*****t*(39.96)=2.62**** *****p*=.012****
One-pattern	CT: 2.05 ± .22 CON: 2.19 ± .51	*t*(27.04)=-1.78,*p*=.25	CT: 2.00 (.000) CON: 2.00 (.000)	–
Two-pattern	CT: 2.76 ± .83 CON: 3.29 ± 2.41	*t*(24.68)=-.94,*p*=.36	CT: 2.19 ± .40 CON: 3.33 ± 2.20	***t*(21.34)=-2.34*p*=.029**
Three-pattern	CT: 5.24 ± 2.36 CON: 5.19 ± 2.84	*t*(40)=.06 *p*=.95	CT: 3.48 ± 2.40 CON: 5.14 ± 3.01	*t*(40)=-1.99 *p*=.054
Six-pattern	CT: 6.62 ± 3.47 CON: 6.62 ± 3.25	*t*(40)=.001 *p*=1.00	CT: 5.57 ± 3.49 CON: 7.14 ± 3.17	*t*(40)=-1.53 *p*=.13
Eight-pattern	CT: 8.52 ± 2.29 CON: 8.14 ± 2.82	*t*(40)=.48 *p*=.63	CT: 6.57 ± 3.50 CON: 7.95 ± 3.04	*t*(40)=-1.37 *p*=.18

*Notes:* CT: Cognitive Training group; CON: Control group

##### Trials.

The time-by-pattern-by-group interaction for total trials adjusted was significant [*F*(4,37)=3.08, *P*=.027, η_p_^2^=.25] with main effects of time [*F*(1.40)=7.42, *P*=.01, η_p_^2^=.16] and pattern [*F*(4,37)= 47.00, *P*<.001, η_p_^2^=.84] (Figure 2). A follow-up independent samples t-test showed that the Cognitive Training group needed significantly fewer trials at the second-pattern stage compared with the control group at outcome [*t*(21.34)=-2.34, *P*=.024, d=.63] (Table 2). The difference in means for the number of trials needed between groups at the third-pattern stage at outcome did not reach significance [*t*(40)=-1.99, *P*=.054]. Paired samples *t* tests showed that the number of trials needed from baseline to outcome was significantly reduced at the second [*t*(20)=3.23, *P*=.004] and third [*t*(20)=2.44, *P*=.024] pattern stages in the cognitive training group, but not the control group (*P*>.92). Total trials were also significantly reduced within the cognitive training group [*t*(20)=3.72, *P*=.001] but not the control group [*t*(20)=-.11, *P*=.91]. Due to the potential confounding effects of anxiety and depression on memory in individuals with MCI, PAL analyses were repeated using baseline HADS score as a covariate. Key Time-by-Pattern-by-Group interactions remained and were: errors, *F* = 2.64, *P* = .049 and trials, *F* = 3.15, *P* = .026.

##### First Trial Memory Score. 

The time-by-group interaction for first trial memory score was also significant [*F*(1,40)=10.56, *P*=.002, η_p_^2^=.21], with a main effect of time [*F*(1.40)=9.04, *P*=.005, η_p_^2^=.18 (Figure 2). A follow-up independent samples *t* test showed that group means significantly differed at outcome [*t*(39.96)=2.62, *P*=.012, d=.83] (Table 2). Paired samples *t* tests showed that first trial memory scores were significantly improved from baseline to outcome in the cognitive training group [*t*(20) =-4.21, *P*<.001] but not the control group [*t*(20)=.18, *P*=.86].

**Figure 3.**
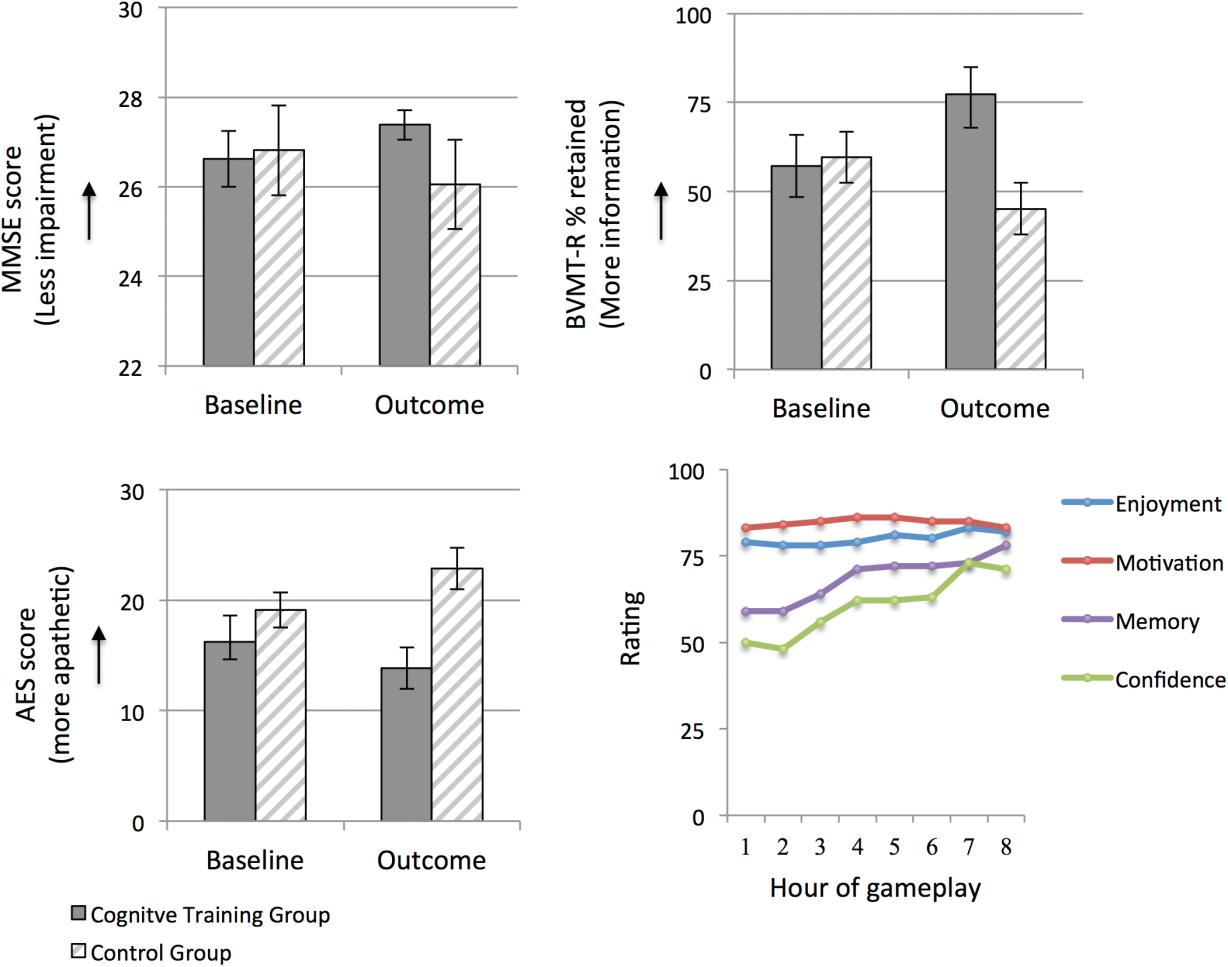
Scores on the Mini Mental State Examination (MMSE), percent of information retained on the Brief Visuospatial Memory Test-Revised (BVMT-R), and scores on the Apathy Evaluation Scale (AES) by group at baseline and outcome. Individuals in the cognitive training group maintained high levels of motivation and enjoyed playing the game; self-confidence improved over the course of the cognitive training sessions.

**BVMT-R—**The time-by-group interaction for the percentage of information retained during the recognition phase of the BVMT-R was significant [*F*(1,37)=4.95, *P*=.032, η_p_^2^ =.12 (percentage retained at baseline: cognitive training group mean=57.2%, SD=40.0, control group mean=59.63%, SD=38.0; percentage retained at outcome: cognitive training group mean=77.2%, SD=34.3, control group mean=45.2%, SD=40.9] (Figure 3). Group means significantly differed at outcome [*t*(37)=2.66, *P*=.012, d=.85] but not baseline [*t*(38)=-.20, *P*=.84]. However, within-group comparisons from baseline to outcome were not significant for either group [cognitive training: *t*(19)=-1.95, *P*=.07; control group: *t*(18)=1.25, *P*=.23]. The interactions reflecting total recall, learning, and delayed recall were not significant (all *P*>.11).

**MMSE—**The time-by-group interaction for the MMSE was significant [*F*(1,40)=4.70, *P*=.036, η_p_^2^=.11 (MMSE at baseline: cognitive training group mean=26.6, SD=2.9, control group mean=26.8, SD=2.2; MMSE at outcome: cognitive training group mean=27.4, SD=1.5, control group mean=26.1, SD=2.4] (Figure 3). Group means significantly differed at outcome [*t*(40)= 2.15, *P*=.038, d=.66, but not baseline [*t*(40)=-.24, *P*=.81]. However, within-group comparisons from baseline to outcome were not significant for either group [cognitive training group: *t*(20)=-1.75, *P*=.09; control group: *t*(20)=1.38, *P*=.18].

**Neuropsychiatric Symptoms—**The interaction reflecting GDS scores did not reach significance [*F*(1,40)=3.59, *P*=.074]. A significant time-by-group interaction was found for the AES [*F*(1,40)=5.32, *P*=.026, η_p_^2^=.12 (AES at baseline: cognitive training group mean=16.2, SD=10.8, control group mean=19.1, SD=7.4; AES at outcome: cognitive training group=13.9, SD=8.5, control group=22.9, SD=8.7] (Figure 3). Group means significantly differed at outcome [*t*(40)=-3.40, *P*=.002, d=1.05] but not baseline [*t*(40)=-1.00, *P*=.32] (Figure 3). Paired samples *t* tests showed that apathy was significantly increased from baseline to outcome in the control group [*t*(20)=-2.16, *P*=.04] but not the cognitive training group [*t*(20)=1.18, *P*=.25].

**CANTAB CRT—**The time-by-group interactions for the percentage of correct trials and mean latency (i.e., response speed) for correct trials were not significant [*F*(1,40)=.84, *P*=.37] and [*F*(1,40)=1.29, *P*=.26, respectively (percentage of correct trials at baseline: cognitive training group mean=98.4%, SD=2.8]. Control group mean=94.7%, SD=21.5; percentage of correct trials at outcome: cognitive training group mean=98.4%, SD=1.6, control group mean=99.1%, SD=1.9; latency at baseline: cognitive training group mean=542.8, SD=197.9, control group mean=469.5, SD=157.7; latency at outcome: cognitive training group mean=541.0, SD=187.6, control group mean=512.6, SD=192.5). Thus, the 2 groups did not differ in terms of alertness or general motor speed.

**VAS after Gameplay—**High levels of enjoyment and motivation were maintained throughout all hours of gameplay (all ratings >.76), whereas self-confidence and self-reported memory ability improved over time (range from .48 to .78) (Figure 3). All participants in the cognitive training group completed every hour of gameplay and no participant withdrew from the study.

## Discussion

We tested a novel learning and memory game for use on an iPad including its effects on cognition and motivation in patients with aMCI. We hypothesized that patients with aMCI would show improved episodic memory and visuospatial abilities after 8 hours of cognitive training compared with a control group that attended clinic as usual. Due to the gamification of our cognitive training program, we further hypothesized that high levels of enjoyment and motivation would be maintained after each hour of gameplay.

### Effects of Cognitive Training on Episodic Memory and Visuospatial Abilities

In support of our main hypothesis, cognitive training with Game Show led to improvements in episodic memory as measured by the CANTAB PAL task. Effect sizes found for total errors (23%), total trials (25%), and first trial memory score (21%) were modest between groups, but larger than for all other outcome measures. Specifically, the cognitive training group showed significantly better performance compared with the control group in terms of reduced errors and the number of trials needed for completion. For errors, the cognitive training group showed better performance at the second- and third-pattern stages. For trials, the cognitive training group showed better performance at the second-pattern stage. The cognitive training group also correctly remembered the locations of more patterns after the first trial summed across all stages completed (first trial memory score), which was the only significant between-group interaction that would survive strict correction for multiple comparisons. However, within-group comparisons revealed highly specific effects of cognitive training on episodic memory. Here, patients showed meaningful effects of training at *lower* loads of PAL task difficulty. Improvements at easier pattern stages reflect previous work from our laboratory showing that hippocampal activation is dependent upon memory load, such that patients with MCI activate more at lower loads and less at higher loads compared with controls (de Rover et al., 2011). A differential pattern of activation suggests that cognitive interventions for hippocampal-dependent memory deficits may be most effective at more tractable levels of PAL task difficulty. Furthermore, impaired performance on this task is known to be sensitive and specific for the early and differential diagnosis of AD (Swainson et al., 2001; Blackwell et al., 2004), with increased errors associated with decreased hippocampal volume (Nathan et al., 2017). PAL performance is therefore one candidate neuropsychological marker for preclinical AD (Beddington et al., 2008). Importantly, our study demonstrates that cognitive training robustly improves PAL task performance within patients with aMCI, emphasizing that memory deficits are not necessarily stable and are amenable to change early in the course of the neurodegenerative process. These results are also consistent with our previous study showing that patients with schizophrenia demonstrate improved episodic memory following cognitive training with a memory game on an iPad (Sahakian et al., 2015).

Results from the BVMT-R showed that the cognitive training group retained significantly more complex geometric information at delayed recall compared with the control group at outcome. This was anticipated, as both our memory game and the BVMT-R require participants to memorize different geometric patterns in a given spatial location. Data from this task suggests important convergent validity that our game produces an effect on an independent, but conceptually related, measure of visuospatial memory. Although encouraging, this result requires confirmation in a larger sample, as improved visuospatial abilities within a cognitive training group would substantiate training effects with evidence of near transfer.

### Effects of Cognitive Training on General Cognition and Neuropsychiatric Symptoms

Changes in MMSE scores were unexpected. Scores in both groups changed by a difference of more than half a point (higher for the cognitive training group, and lower for the control group) and significantly differed at outcome. However, MMSE scores from baseline to outcome were not significantly different within either group. Given that our training program specifically targeted episodic memory, it is possible that the trained cognitive domain would have generalized to improvements at the global level. However, a recent meta-analysis concluded that cognitive stimulation of multiple domains, typically including a social component, is the only cognitive intervention that improves general cognition in dementia (Huntley et al., 2015). We therefore interpret our significant interaction with caution. Execution of our memory game in a group format, or introduction of an opponent or companion player option, might increase socialization needed to achieve these effects. This would be most advantageous if delivered before dementia is already diagnosed, as damage to the brain is usually too severe for improvements in global outcome measures following cognitive intervention in later stages of disease progression.

Lastly, apathy significantly increased within the control group from baseline to outcome. This may be due to indifference associated with knowledge about participation in the nonintervention group. Apathy scores from baseline to outcome did not significantly differ within our cognitive training group, but no participant showed clinically significant apathy at either time point. Use of gaming technology to reduce apathetic symptoms, however, has shown promise. “Serious” gaming, for example, has been successfully adapted for use in MCI patients with high levels of apathy (Manera et al., 2015). Cognitive stimulating activities have also been shown to reduce apathetic and depressive symptoms in older adults with memory complaints (Buettner et al., 2011). In addition to increasing the motivational aspects of cognitive training, serious games also have the potential to target impaired emotional and social processes, which would be beneficial for reducing neuropsychiatric symptoms in patients with aMCI and AD.

### Motivational Enhancement

One of the key aims of our study was to ensure that Game Show was developed with patient feedback to be easily understood, highly enjoyable, and motivating. Use of an iPad allowed for the titration of difficulty depending on individual performance, thus taking a more personalized approach to keep users engaged. Our game was further designed to have the same motivational properties typical of computer games, including stimulating music, character assistance, opportunities for progression, feedback and humor, in an effort to maximize player engagement and circumvent dropout rates often problematic of cognitive training studies (Sahakian et al., 2015). Most cognitive training programs are overly repetitive and do not address the motivational deficits associated with older populations with memory difficulties (Savulich et al., 2017). We observed that all participants in the cognitive training group completed each hour of gameplay and, importantly, reported high levels of enjoyment and motivation to continue, with self-confidence and self-rated memory ability improving over time. The lack of dropout in our study represents a motivational improvement over traditional cognitive training programs, where the attrition rate can be >15% (Wykes et al., 2011) and encourages the potential for a wider application of serious games for cognitive training in other clinical populations with severe motivational deficits, such as traumatic brain injury or remitted depression.

### Limitations

Our study had limitations. Firstly, our sample size was relatively small, with limited statistical power. Secondly, we did not include an active control group, such as patients with aMCI playing a nonmemory-related game or a game with no specific cognitive content. Although we cannot preclude the possibility that the effects observed here were epiphenomena of touch screen technology use more generally, we consider this unlikely as both groups reported similar levels of computer gaming, Internet use, and confidence using new technology at baseline. It is also possible that increased contact time and interaction with the research team, particularly with the elderly, positively impacts cognitive performance, raises self-esteem, or offers some other confounding social benefit not controlled for here. Replication and extension with a larger, more controlled trial, including an active control group with access to an iPad, but not our memory game, would address these methodological shortcomings. Although not within the scope of the current study, the duration to which cognitive improvements persist and their dependence on continued gameplay pose important areas of future research.

### Strengths

Strengths of our study include in depth public and patient involvement during the development of our game, the likelihood that portable devices increase active engagement with participation in cognitive training, and the reduction in negative stigma since games are popular with healthy people of all ages. Gamified cognitive training is also cost effective and not associated with side effects inevitably reported by patients taking medication. Integrating findings from neuropsychiatry with gaming technology could also lead to more widespread use of innovative nonpharmacological strategies for cognitive restoration and enhancement and help alter public perception of cognitive interventions for memory (Sahakian et al., 2015; Savulich et al., 2017).

## Conclusions

Overall, the present study demonstrates that episodic memory is one cognitive process that can be targeted, with success, for enhancement by gamified cognitive training in patients with aMCI. Importantly, cognitive training with a game effectively maintains high levels of participant enjoyment and motivation and promotes confidence both in the self and subjective memory ability. Cognitive training also has the potential to improve visuospatial abilities. Early intervention and effective treatment is critical for neurodegenerative disorders, particularly before extensive neuropathological damage (Beddington et al., 2008; Sahakian et al., 2010; Collins et al., 2011). Novel symptomatic interventions are urgently needed, and recent interest in nonpharmacological approaches of enhancement, which utilize novel technologies to target cognitive symptoms rather than diagnostic category, offer an alternative means for improving cognition in the absence of effective drug treatments (Insel and Sahakian, 2012; Insel et al., 2013; Sahakian, 2014). Gamified cognitive training strategies could thus be used to ameliorate memory deficits and increase motivation, either independently or in combination with other interventions for the elderly, such as physical exercise or pharmacological treatments, to synergize the effects needed for good cognitive outcome for patients with aMCI and mild AD. Larger, more controlled trials are required to test these hypotheses.

## Funding

This work was supported by a grant from Janssen Pharmaceutica/Johnson & Johnson (71418) awarded to Barbara J. Sahakian and the NIHR Biomedical Research Centre (Neurodegeneration; Mental Health), Cambridge. James B. Rowe is supported by the Wellcome Trust (103838). George Savulich is currently funded by a grant from Eton College and the Wallitt Foundation.

## Statement of Interest

George Savulich and Thomas Piercy consult for Peak. Chris Fox has received speaker fees from Astellas Pharmaceuticals. John Suckling has acted as a consultant for GlaxoSmithKline. James B. Rowe has received research grants from AZ-Medimmune and Janssen. John T. O’Brien has acted as a consultant for TauRx, Axona, and Lilly. Barbara J. Sahakian consults for Cambridge Cognition, Peak and Mundipharma.

## References

[CIT0001] Albert MS DeKosky ST Dickson D Dubois B Feldman HH Fox NC Gamst A Holtzman DM Jagust WK Petersen RC Snyder PJ Carrillo MC Thies B Phelps CH (2011) The diagnosis of mild cognitive impairment due to Alzheimer’s disease: recommendations from the National Institute on Aging and Alzheimer’s Association workgroup. Alzheimers Dement 7:270–279. 21514249 10.1016/j.jalz.2011.03.008 PMC3312027

[CIT0002] Anguera JA Boccanfuso J Rintoul JL Al-Hashimi O Faraji F Janowich J Kong E Larraburo Y Rolle C Johnston E Gazzaley A (2013) Video game training enhances cognitive control in older adults. Nature 501:97–101. 24005416 10.1038/nature12486 PMC3983066

[CIT0003] Barnett JH Blackwell AD Sahakian BJ Robbins TW (2016) The Paired Associates Learning (PAL) test: 30 years of CANTAB Translational Neuroscience from Laboratory to Bedside in Dementia Research. In: Current topics in behavioral neuroscience: translational neuropsychopharmacology (Robbins TW Sahakian BJ, eds), vol. 28, pp. 449–474. 10.1007/7854_2015_5001 27646012

[CIT0004] Beddington J Cooper CL Field J Goswami U Huppert FA Jenkins R Jones HS Kirkwood TB Sahakian BJ Thomas SM (2008) The mental wealth of nations. Nature 455:1057–1060. 18948946 10.1038/4551057a

[CIT0005] Belleville S (2008) Cognitive training for persons with mild cognitive impairment. Int Psychogeriatr 20:57–66. 17958927 10.1017/S104161020700631X

[CIT0006] Benedict RH (1997) Brief visuospatial test-revised: professional manual. Odessa, FL: PAR.

[CIT0007] Blackwell AD Sahakian BJ Vesey R Semple JM Robbins TW Hodges JR (2004) Detecting dementia: novel neuropsychological markers of preclinical Alzheimer’s disease. Dement Geriatr Cogn Disord 17:42–48. 14560064 10.1159/000074081

[CIT0008] Brühl A Sahakian BJ (2016) Drugs, games, and devices for enhancing cognition: implications for work and society. Ann N Y Acad Sci 1369:195–217. 27043232 10.1111/nyas.13040

[CIT0009] Buettner LL Fitzsimmons S Atav S Sink K (2011) Cognitive stimulation for apathy for probable early-stage Alzeimer’s. J Aging Res 480890. 21584241 10.4061/2011/480890 PMC3092580

[CIT0010] Collins PY (2011) Grand challenges in global mental health. Nature 475:27–30. 21734685 10.1038/475027a PMC3173804

[CIT0011] DeCarli C (2003) Mild cognitive impairment: prevalence, prognosis, aetiology, and treatment. Lancet Neuol 2:15–21. 10.1016/s1474-4422(03)00262-x 12849297

[CIT0012] de Rover M Pironti VA McCabe JA Acosta-Cabronero J Sergio Arana F Morein-Zamir S Hodges JR Robbins TW Fletcher PC Nestor PJ Sahakian BJ (2011) Hippocampal dysfunction in patients with mild cognitive impairment: a functional neuroimaging study of a visuospatial paired associates learning task. Neuropsychologia 49:2060–2070. 21477602 10.1016/j.neuropsychologia.2011.03.037

[CIT0013] Dickerson BC Sperling RA (2008) Functional abnormalities of the medial temporal lobe memory system in mild cognitive impairment and Alzheimer’s disease: insights from functional MRI studies. Neuropsychologia 46:1624–1635. 18206188 10.1016/j.neuropsychologia.2007.11.030 PMC2760288

[CIT0014] Eagger SA Levy R Sahakian BJ (1992) Tacrine in Alzheimer’s disease. Acta Neurol Scand Suppl 139:75–80. 1414271 10.1111/j.1600-0404.1992.tb04459.x

[CIT0015] Feldman HH (2007) Effect of rivastigmine on delay to diagnosis of Alzheimer’s disease from mild cognitive impairment: the InDDEx study. Lancet Neurol 6:501–512. 17509485 10.1016/S1474-4422(07)70109-6

[CIT0016] Folstein MF Folstein SE McHugh PR (1975) “Mini-mental state”. A practical method for grading the cognitive state of patients for the clinician. J Psychiatr Res 12:189–198. 1202204 10.1016/0022-3956(75)90026-6

[CIT0017] Gates NJ Sachdev PS Fiatarone Singh MA Valenzuela M (2011) Cognitive and memory training in adults at risk of dementia: a systematic review. BMC Geriatr 11:55. 21942932 10.1186/1471-2318-11-55 PMC3191477

[CIT0018] Huntley JD Gould RL Liu K Smith M Howard RJ (2015) Do cognitive interventions improve general cognition in dementia? A meta-analysis and meta-regression. BMJ Open 5:e005247. 10.1136/bmjopen-2014-005247 PMC4390716 25838501

[CIT0019] Insel TR Sahakian BJ (2012) Drug research: a plan for mental illness. Nature 483:269. 22422245 10.1038/483269a

[CIT0020] Insel TR Voon V Nye JS Brown VJ Altevogt BM Bullmore ET Goodwin GM Howard RJ Kupfer DJ Malloch G Marston HM Nutt DJ Robbins TW Stahl SM Tricklebank MD Williams JH Sahakian BJ (2013) Innovative solutions to novel drug development in mental health. Neurosci Biobehav Rev 37:2438–2444. 23563062 10.1016/j.neubiorev.2013.03.022 PMC3788850

[CIT0021] Jean L Bergeron ME Thivierge S Simard M (2010) Cognitive intervention programs for individuals with mild cognitive impairment: systematic review of the literature. Am J Geriatr Psychiatry 18:281–296. 20220584 10.1097/JGP.0b013e3181c37ce9

[CIT0022] Kaser M Deakin JB Michael A Zapata C Bansal R Ryan D Cormack F Rowe JB Sahakian BJ (2016) Modafinil improves episodic memory and working memory cognition in patients with remitted depression: a double-blind, randomized, placebo-controlled study. Biol Psychiatry Cogn Neurosci Neuroimaging 2:115–122. 10.1016/j.bpsc.2016.11.009 PMC5339412 28299368

[CIT0023] Kirchhoff BA Anderson BA Smith SE Barch DM Jacoby LL (2012) Cognitive training-related changes in hippocampal activity associated with recollection in older adults. Neuroimage 62:1956–1964. 22728150 10.1016/j.neuroimage.2012.06.017 PMC3745284

[CIT0024] Li H Li J Li N Li B Wang P Zhou T (2011) Cognitive intervention for persons with mild cognitive impairment: a meta-analysis. Ageing Res Rev 10:285–296. 21130185 10.1016/j.arr.2010.11.003

[CIT0025] Manera V Petit P-D Derreumaux A Orvieto I Romagnoli M Luttle G David R Robert PH (2015) ‘Kitchen and cooking,’ a serious game for mild cognitive impairment and Alzheimer’s disease: a pilot study. Front Aging Neurosci 17:7–24. 10.3389/fnagi.2015.00024 PMC4362400 25852542

[CIT0026] Marin RS (1996) Apathy: concept, syndrome, neural mechanisms, and treatment. Semin Clin Neuropsychiatry 1:304–314. 10320433 10.1053/SCNP00100304

[CIT0027] Martin M Clare L Altgassen AM Cameron MH Zehender F (2011) Cognition-based interventions for healthy older people and people with mild cognitive impairment. Cochrane Database Syst Rev 19:CD006220. 10.1002/14651858.CD006220.pub2 21249675

[CIT0028] Moher D Schulz KF Atlman DG (2001) The CONSORT statement: revised recommendations for improving the quality of reports of parallel-group randomised trails. Lancet 357:1191–1194. 11323066

[CIT0029] Monastero R Mangialasche F Camarda C Ercolani S Camarda R (2009) A systematic review of neuropsychiatric symptoms in mild cognitive impairment. J Alzheimers Dis 18:11–30. 19542627 10.3233/JAD-2009-1120

[CIT0030] Nathan PJ Ying Lim Y Abbott R Galluzzi S Marizzoni M Babiloni C (2017) Association between CSF biomarkers, hippocampal volume and cognitive function in patients with amnestic mild cognitive impairment (MCI). Neuobiol Aging 53:1–10. 10.1016/j.neurobiolaging.2017.01.013 28189924

[CIT0031] Nelson HE (1982) National Adult Reading Test (NART): for the assessment of premorbid intelligence in patients with dementia: test manual. Windsor, UK: NFER-Nelson.

[CIT0032] Petersen RC (2011) Clinical practice. Mild cognitive impairment. N Engl J Med 364:2227–2234. 21651394 10.1056/NEJMcp0910237

[CIT0033] Petersen RC Thomas RG Grundman M Bennett D Doody R Ferris S Galasko D Jin S Kaye J Levey A Pfeiffer E Sano M van Dyck CH Thal LJ (2005) Vitamin E and donepezil for the treatment of mild cognitive impairment. N Engl J Med 352:2379–2388. 15829527 10.1056/NEJMoa050151

[CIT0034] Reijnders J van Heugten C van Boxtel M (2013) Cognitive interventions in healthy older adults and people with mild cognitive impairment: a systematic review. Ageing Res Rev 12:263–275. 22841936 10.1016/j.arr.2012.07.003

[CIT0035] Rosen AC Sugiura L Kramer JH Whitfield-Gabrieli S Gabrieli JD (2011) Cognitive training changes hippocampal function in Mild Cognitive Impairment: a pilot study. J Alzheimers Dis 26:349–357. 21971474 10.3233/JAD-2011-0009 PMC3277842

[CIT0036] Sahakian BJ (2014) What do experts think we should do to achieve brain health? Neurosci Biobehav Rev 43:240–258. 24742821 10.1016/j.neubiorev.2014.04.002

[CIT0037] Sahakian BJ Morris RG Evenden JL Heald A Levy R Philpot M Robbins TW (1988) A comparative study of visuospatial memory and learning in Alzheimer-type dementia and Parkinson’s disease. Brain 111:695–718. 3382917 10.1093/brain/111.3.695

[CIT0038] Sahakian BJ Owen AM Morant NJ Eagger SA Boddington S Crayton L Crockford HA Crooks M Hill K Levy R (1993) Further analysis of the cognitive effects of tetrahydroaminoacridine (THA) in Alzheimer’s disease: assessment of attentional and mnemonic function using CANTAB. Psychopharmacology (Berl) 110:395–401. 7870908 10.1007/BF02244644

[CIT0039] Sahakian BJ (2010) A UK strategy for mental health and wellbeing. Lancet 375:1854–1855. 20511002 10.1016/S0140-6736(10)60817-3

[CIT0040] Sahakian BJ Brühl AB Cook J Killikelly C Savulich G Piercy T Hafizi J Perez J Fernandez-Egea E Suckling J Jones PB (2015) The impact of neuroscience on society: cognitive enhancement in neuropsychiatric disorders and in healthy people. Philos Trans R Soc Lond B Biol Soc 370:20140214. 26240429 10.1098/rstb.2014.0214 PMC4528826

[CIT0041] Savulich G Piercy T Brühl AB Fox C Suckling J Rowe JB O’Brien JT Sahakian BJ (2017) Focusing the neuroscience and societal implications of cognitive enhancers. Clin Pharmacol Ther 101:170–172. 27557349 10.1002/cpt.457

[CIT0042] Sheikh JI Yesavage JA (1986) Geriatric Depression Scale (GDS): recent evidence and development of a shorter version. Clinical gerontology: a guide to assessment and intervention, pp165–173, New York: The Haworth Press.

[CIT0043] Simon SS Yokomizo JE Bottino CM (2012) Cognitive intervention in amnestic mild cognitive impairment: a systematic review. Neurosci Biobehav Rev 36:1163–1178. 22322184 10.1016/j.neubiorev.2012.01.007

[CIT0044] Strenziok M Parasuraman R Clarke E Cisler DS Thompson JC Greenwood PM (2014) Neurocognitive enhancement in older adults: comparison of three cognitive training tasks to test a hypothesis of training transfer in brain connectivity. Neuroimage 85:1027–1039. 23933474 10.1016/j.neuroimage.2013.07.069

[CIT0045] Swainson R Hodges JR Galton CJ Semple J Michael A Dunn BD Iddon JL Robbins TW Sahakian BJ (2001) Early detection and differential diagnosis of Alzheimer’s Disease and depression with neuropsychological tasks. Dement Geriatr Cogn Disord 12:265–280. 11351138 10.1159/000051269

[CIT0046] Winblad B Gauthier S Scinto L Feldman H Wilcock GK Truyen L Mayorga AJ Wang D Brashear HR Nye JS (2008) Safety and efficacy of galantamine in subjects with mild cognitive impairment. Neurology 70:2024–2035. 18322263 10.1212/01.wnl.0000303815.69777.26

[CIT0047] World Health Organization (April 2016). Dementia Available at http://www.who.int/mediacentre/factsheets/fs362/en/. Accessed January 2, 2017.

[CIT0048] Wykes T Huddy V Cellard C McGurk SR Czobor P (2011) A meta-analysis of cognitive remediation for schizophrenia: methodology and effect sizes. Am J Psychiatry 168:472–485. 21406461 10.1176/appi.ajp.2010.10060855

[CIT0049] Zigmond AS Snaith RP (1983) The hospital anxiety and depression scale. Acta Psychiatr Scand 67:361–370. 6880820 10.1111/j.1600-0447.1983.tb09716.x

